# Implementing an Exercise Physiology Clinic for Consumers Within a Community Mental Health Service: A Real-World Evaluation

**DOI:** 10.3389/fpsyt.2021.791125

**Published:** 2021-11-24

**Authors:** Hamish Fibbins, Law Edwards, Rachel Morell, Oscar Lederman, Philip Ward, Jackie Curtis

**Affiliations:** ^1^Keeping the Body in Mind, Sydney, NSW, Australia; ^2^School of Psychiatry, UNSW Sydney, Randwick, NSW, Australia; ^3^Mindgardens Neuroscience Network, Sydney, NSW, Australia; ^4^Schizophrenia Research Unit, Liverpool Hospital, Ingham Institute of Applied Medical Research, Sydney, NSW, Australia

**Keywords:** physical activity, exercise, mental illness, interventions, exercise specialists, exercise physiologists, mental health

## Abstract

**Background:** Physical activity significantly improves mental illness symptoms and physical health for people living with mental illness. Mental health services do not routinely provide their consumers with access to exercise professionals for physical activity engagement. Barriers exist to integrating physical activity as part of standard care including staff culture, finance, and resources. This study examines the feasibility of newly established exercise physiology clinic within a mental health service in Sydney, Australia.

**Methods:** A single site, open trial was conducted in a community centre within a large mental health district. A meeting room was converted into a part-time exercise physiology clinic where individualised physical activity interventions were delivered by an accredited exercise physiologist. Outcome measures including BMI, cardiovascular fitness, and self-reported physical activity were collected.

**Results:** A total of 84 mental health consumers (17% of eligible consumers within the mental health service) participated in the clinic on average for one exercise session weekly. Moderate-to-vigorous physical activity significantly increased and sedentary time significantly decreased (*p* < 0.001).

**Conclusions:** Exercise physiology clinics are feasible within mental health services and should be incorporated as part of standard care.

## Introduction

People with severe mental illness face a reduced life expectancy of 15–20 years compared to the general population (1); this is overwhelmingly attributed to chronic physical illnesses such as cardiovascular disease and diabetes (2). While side effects from antipsychotic medications contribute largely to this higher incidence of cardiometabolic disease (3), modifiable lifestyle risk factors also impact significantly. High rates of tobacco smoking (4), physical inactivity (5), poor nutrition (6) and low cardiorespiratory fitness (7) are prevalent for people with severe mental illness and influence the widening life-expectancy gap (1) while contributing to the global burden of disease (8).

Lifestyle interventions, incorporating nutrition and physical activity, are beneficial in reducing cardiometabolic risk (5, 6, 9) whilst also having positive effects on mental health symptomatology, cognition and psychosocial functioning for people with severe mental illness (10, 11). Evidence suggests that such lifestyle interventions are most effective when delivered by experts in the relevant fields, including dietitians and exercise physiologists, as they can adopt evidence-based practises and utilise behaviour change techniques to maximise long term adherence in people with severe mental illness (6, 12–14). For instance, dropout rates are lower and adherence is improved when exercise professionals deliver physical activity interventions to people with mental illness (15, 16).

A 2019 Lancet commission called for mental health services to include lifestyle interventions, including physical activity, to protect the physical health of people with severe mental illness (17). Calls to action for implementation and integration of lifestyle programs in routine mental health care have been made by Australian and international peak exercise professional and psychiatric organisations (12, 18–20). It is important to establish the feasibility of lifestyle interventions in community mental health settings, with a focus on real-world conditions (21–23). Evaluating such novel strategies would increase widespread adoption of these programs by mental healthcare systems (24).

In this paper we evaluated the feasibility of a novel exercise physiology clinic delivered as routine care to people living with severe mental illness receiving treatment in a community mental health centre. With a focus on delivering physical activity interventions within real-world conditions, a newly created exercise physiology clinic was conceived, implemented, and adopted as part of standard mental health care.

## Methods

The study was assessed by the South Eastern Sydney Local Health District (SESLHD) Human Research Ethics Committee and was determined to be a quality improvement or quality assurance activity not requiring independent ethics review [17/298(LNR/17/POWH/580)].

### Study Design

A single site, open trial was conducted in a community centre located within a large public health district providing mental health services to ~600 people living with severe mental illness. The community centre, while linked to a major teaching hospital, is sited in the local community, and comprised a waiting room, consumer appointment rooms, staff offices, and a large meeting room. People living with severe mental illness who access this centre are provided a package of care consisting of support by a multidisciplinary team. This team includes psychiatrists, mental health nurse specialists, occupational therapists, clinical psychologists, and social workers. Collectively, the mental health team supported consumers through a combination of pharmacotherapy and psychosocial interventions which utilise person-centred and recovery-oriented practises. In addition to the mental health team, the community centre has a physical health team. The *Keeping the Body in Mind* team was comprised of a clinical nurse consultant, accredited practising dietitian, accredited exercise physiologist, and a peer worker with lived experience of mental illness. *Keeping the Body in Mind* initially was established as a pilot programme and demonstrated antipsychotic-related weight gain in first episode psychosis in those aged 14–25 could be attenuated through lifestyle interventions (25, 26). Following this, *Keeping the Body in Mind* teams were deployed by the mental health service to address the physical health needs of youth and adult consumers through a combination of individualised and group-based lifestyle interventions.

When the *Keeping the Body in Mind* team began working at the community health centre, no exercise facilities existed, and physical activity interventions could not be performed on-site, significantly limiting the scope of exercise physiology services. Consumers accessed individual consultations with the accredited exercise physiologist to discuss their physical activity as well as engaging in motivational interviewing and goal setting. Following an allocation of funding from the mental health service to purchase small items of equipment, and discussions with centre management, the large meeting room was converted to a “make-shift” exercise physiology clinic during times when meetings did not occur.

All adult community case managed consumers were eligible for the exercise physiology clinic. Recruitment to the clinic occurred via referrals from staff members who managed the mental health services provided to consumers.

### Evolution of the Clinic and Physical Activity Intervention

Under the direction of the accredited exercise physiologist, mental health consumers were initially able to attend the clinic (located within the staff meeting room) one day each week which increased to three days a week as awareness and interest from both consumers and staff increased. Due to limited funding, only a small amount of equipment could be purchased, including a stationary bicycle and a set of dumbbells. Over the next three months, more equipment was acquired with additional funding from the mental health service, including a rowing machine, and boxing and bench-press equipment.

Upon referral, consumers had an initial consultation with the accredited exercise physiologist, to obtain baseline measures, discuss physical activity goals and engage in motivational interviewing. An initial physical activity program and plan was developed in this session. Consultations with the accredited exercise physiologist were subsequently provided as required, typically monthly, with outcomes measures repeated every three months. More frequent consultations could occur if requested.

To align exercise physiology interventions with recovery-oriented mental health treatment approaches and to support engagement in the clinic, the physical activity programs were client-centred, i.e., tailored to the consumers' interests, goals, level of motivation, readiness to change, and physical capabilities. Consumers could engage in a range of activities including, but not limited to, aerobic exercise on machines, strength training or boxing for fitness. Yoga sessions and Zumba dance classes directed by “YouTube” instructional videos, and supervised by the accredited exercise physiologist, were also offered. Mental health peer workers employed by the mental health service were engaged in the referral process and ongoing to assist participating consumers in navigating the exercise service.

Consumers could attend the clinic as often they wished during business hours, typically booking in to 30–60-min timeslots. As clinic attendance increased, final year university exercise physiology students on clinical placement were incorporated into the clinic, assisting those attending with exercise prescription, including modifying the program, providing instruction, demonstration, and feedback.

### Outcome Measures

Baseline measures were collected during the initial consultation with the accredited exercise physiologist and repeated at least on a 3-monthly basis for the duration of the consumer engagement in the service. Outcome measures included:

#### Anthropometry

Height and weight, were used to calculate Body Mass Index (BMI) using standardised procedures. Participants were weighed without shoes and wearing light clothing on the OMRON HN-283 digital scale to the nearest 0.1 kg. Height was measured with shoes off, using a wall-mounted stadiometer to the nearest 0.1 cm. BMI was calculated as weight (kg)/height (m)^2^ with participants characterised as normal weight (18.5–24.9 kg/m^2^), overweight (25–29.9 kg/m^2^), obese (30–39.9 kg/m^2^) and morbidly obese (≥40 kg/m^2^) according to World Health Organisation criteria (27).

#### Cardiorespiratory Fitness

This was assessed via the Astrand Rhyming submaximal test (28). The test involves 6-min of cycling on a cycle ergometer (Monark 828E Ergomedic bike) at a specified resistance whilst measuring heart rate response. The average heart rate of the final two min of exercise is recorded and used via the Astrand-Rhyming gender-sensitive nomogram to estimate the participants' VO_2_max, or maximum rate of oxygen consumption, an indicator of cardiorespiratory fitness. Results were then normalised to age using the Astrand-Rhyming age-correction factor (29).

#### Self-Reported Physical Activity

This was assessed using questions taken from the short-form International Physical Activity Questionnaire (30). Questions refer to activity performed over the previous seven days and categorises activity according to vigorous activities, moderate activities, walking and sedentary time. Vigorous and moderate physical activity levels were combined for analysis to reflect current Australian physical activity guidelines (31).

### Statistical Analysis

Data analysis was conducted using the Statistical Package for the Social Science, Version 27.0. Outcome measures were assessed using paired sample *t*-tests with a Bonferroni's correction applied for multiple statistical tests. For paired sample *t*-tests using a Bonferroni's correction results were significant if *p* ≤ 0.01.

## Results

Participant demographic information and clinic attendance data is presented in Table 1. During the period the exercise physiology clinic was operating, there were 483 individuals who were eligible for referral. In total, *n* = 84 consumers (17% of eligible consumers) attended the exercise physiology clinic between May 2018 and March 2020. Just over half of participants were male (*n* = 43, 51%) and the largest cohort were between the ages of 46 and 55 (*n* = 28, 33%). The majority had a diagnosis of schizophrenia or schizoaffective disorder (*n* = 75, 89.3%) and almost all had been prescribed antipsychotic medication (*n* = 86, 98%). Of the consumers prescribed antipsychotic medication, 47 (56%) took one form of oral antipsychotic medication and 11 (13%) took more than one type of oral antipsychotic medications. There were 9 (11%) consumers who received one type of antipsychotic medication via a depot injection and 15 (18%) were prescribed both an oral and a depot injection antipsychotic medication. Of the consumers who were taking oral antipsychotic medication, 31 were prescribed Clozapine (35.2) 7 (8%) were prescribed Olanzapine.

**Table 1:** Participant demographics, clinic attendance (*n* = 84).

**Age Categories**	n (%)	
18–25		6 (7.1)
26–35		12 (14.3)
36–45		23 (27.4)
46–55		28 (33.3)
55+		15 (17.9)
**Mental Health Diagnosis**	n (%)	
Schizophrenia		51 (60.7)
Schizoaffective Disorder		24 (28.6)
Bipolar Disorder		7 (8.3)
First Episode Psychosis		1 (1.2)
Major Depressive Disorder		1 (1.2)
**Number of Exercise Physiology** **Clinic Sessions Attended**	n (%)	
1 session		13 (15.5)
2–5 sessions		22 (26.2)
6–10 sessions		17 (20.2)
11–20 sessions		10 (11.9)
>20 sessions		22 (26.2)
**Time spent in the Exercise Physiology Clinic**	n (%)	
<1 month		16 (19.0)
1–3 months		18 (21.4)
3–6 months		11 (13.1)
>6 months		39 (46.4)

In total, *n* = 39 (46%) of participants attended the exercise physiology clinic for a period of 6 months or more and the mean number of sessions attended was 17 (SD ± 22.9). Participants attended the clinic on average one session a week (SD ± 1.3). There were 16 participants (19%) that engaged with the clinic for <1 month.

Results of the statistical analysis of outcome measures are presented in Table 2. Upon commencement of the clinic, participants had a mean baseline BMI of 30.9 (SD ± 7.1), placing the average participant in the “obese” BMI category. In total, physical activity data was collected for *n* = 61 (73%) of participants on commencement at the clinic. Of these participants, the mean self-reported weekly moderate to vigorous physical activity level was 71 min (SD ± 100.7) and as such, *n* = 49 participants (81% of participants with completed physical activity follow-up data) did not meet the Australian recommended guidelines for physical activity of at least 150 min of moderate to vigorous intensity exercise per week (31).

**Table 2:** Pre- and post-outcome measures.

	**N**	**Baseline** **mean (SD)**	**Post mean** **(SD)**	**Mean difference** **(95% CI)**	**T value**	**df**	***P*** **value** **(two-tailed)**
MVPA (min/week)	29	88.1 (108.8)	162.3 (139.7)	74.2 (31.92, 116.57)	3.59	28	0.001
Walking (min/week)	29	163.8 (245.8)	205.2 (205.7)	41.4 (−16.73, 99.49)	1.50	28	0.156
Sitting (min/day)	29	5.9 (2.1)	4.4 (2.3)	−1.6 (−2.4, 0.71)	−3.72	28	0.001
BMI	55	30.92 (7.1)	30.90 (7.2)	−0.01 (−0.8, 0.9)	0.03	54	0.977
Vo2max	32	23.8 (6.9)	25.5 (8.2)	1.7 (−0.4, 3.8)	−1.61	31	0.117

In total, *n* = 55 participants (65%) completed follow-up anthropometry measurements. Weight neutrality was observed with no statistically significant changes to participants' BMI during the program (mean change = −0.01, 95% CI −0.88, 0.86; *t* = −0.03, *p* = 0.98).

**Figure 1:**
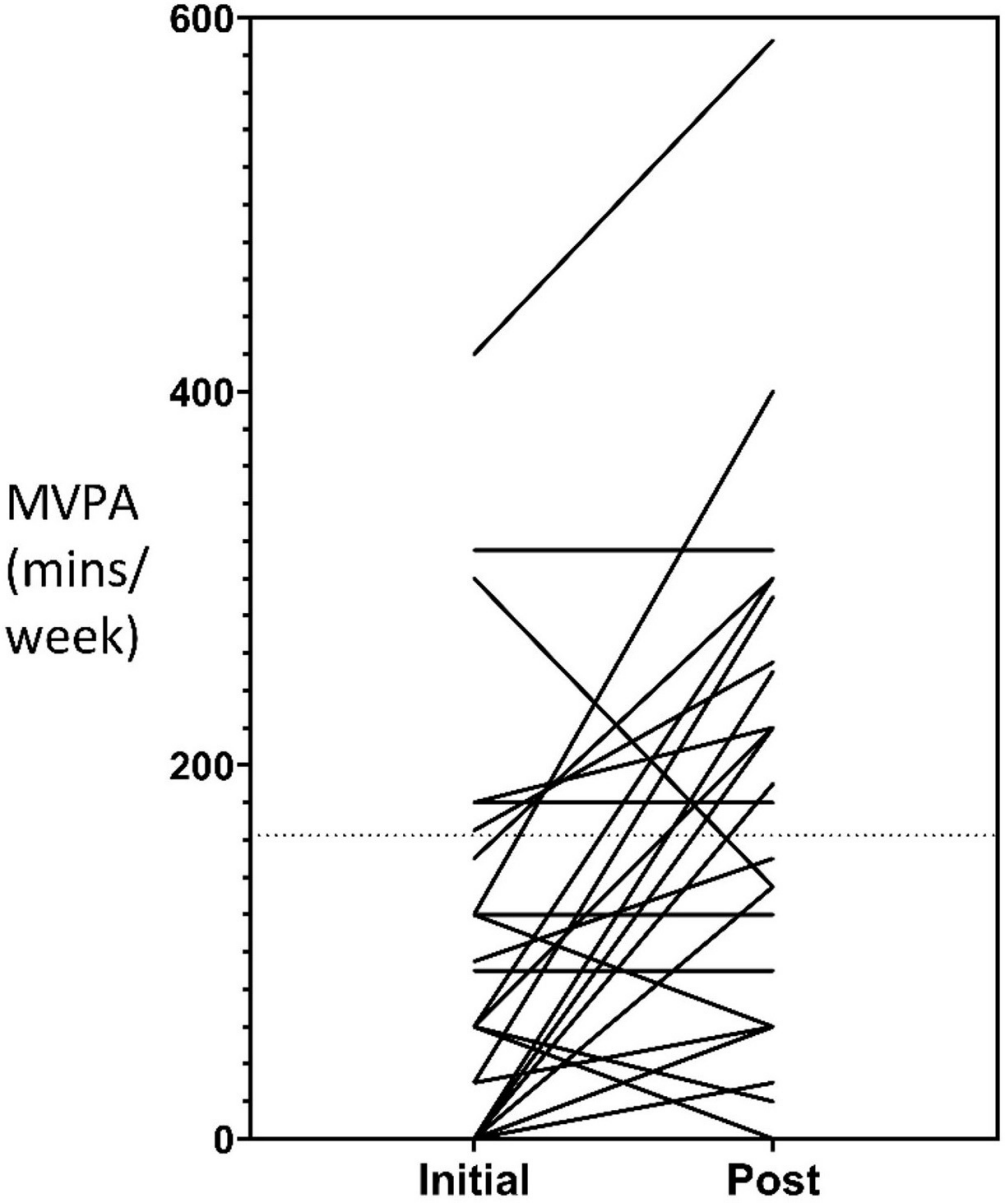
Moderate-vigorous physical activity pre- and post-values.

**Figure 2:**
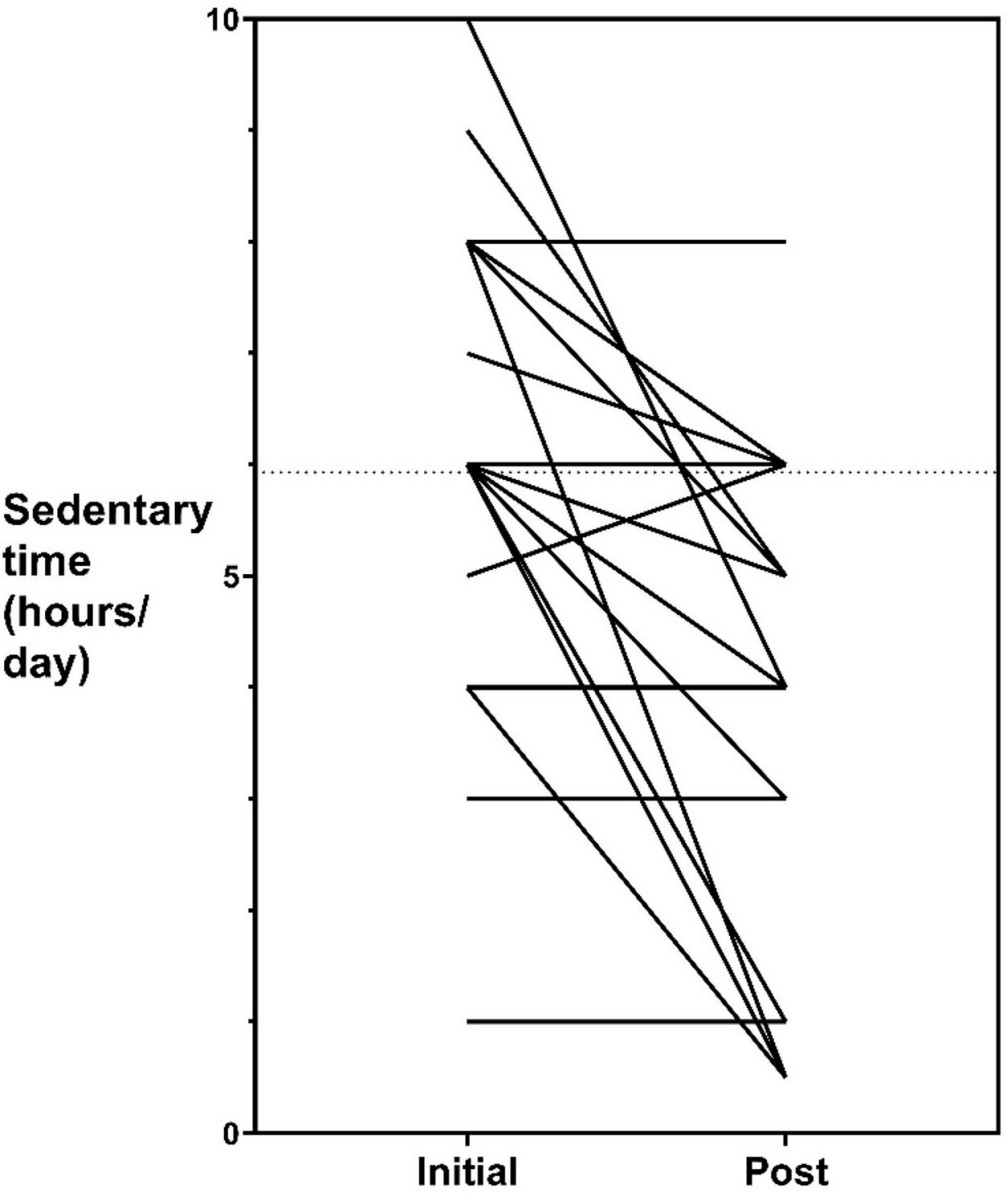
Sedentary time pre- and post-values.

Follow-up cardiovascular fitness measures were collected for *n* = 32 participants (38%) and follow-up self-reported physical activity measures were collected for *n* = 29 participants (35%). The mean time between baseline and follow-up fitness and physical activity measures was 333 days (SD ± 180, range = 42–581) and 347 days (SD ± 178, range = 42–623) respectively. A mean increase to moderate-to-vigorous physical activity of 74-min per week occurred for participants during their involvement in the service which represented statistically significant changes (95% CI 31.9, 116.6; *t* = 3.6, *p* < 0.001). Of the *n* = 29 participants who completed follow-up measures, the number that met the Australian recommended guidelines for physical activity of at least 150 min of moderate to vigorous intensity exercise per week, increased from 7 (24.1%) to 14 (48.3%). A mean decrease to daily sedentary time of 1.6 h occurred which represented statistically significant changes (95% CI −2.4, 0.71; *t* = −3.7, *p* < 0.001). No statistically significant changes occurred for cardiovascular fitness and time spent walking (both *p* values > 0.01). Individual changes to participant's moderate to vigorous physical activity levels and sedentary time is represented graphically in Figures 1, 2, respectively.

## Discussion

Our findings demonstrated that a new mental health exercise physiology clinic conducted under real-world conditions was feasible for people living with severe mental illness. Feasibility of an exercise physiology service in a recent study for youth at risk of mental illness and attendance was deemed “well-attended” at ~1 session per week (32). In addition to the clinic in our study being well-attended, engagement in higher levels of moderate-to-vigorous physical activity and a decrease in sedentary time occurred for participants following their involvement. Despite this, no significant improvements were evident in cardiovascular fitness or BMI throughout the program. In the absence of a control comparison group, inferences regarding the association between changes in outcome measures and clinic attendance should be made with caution.

Given the non-significant changes to fitness and anthropometry measures despite regular clinic attendance, it is important to consider the possible reasons underpinning these findings. Perhaps the weekly exercise sessions did not produce substantial physiological changes in fitness measures, without participants also engaging in additional home-based exercise. Additionally, as body weight is predominantly impacted by nutrition, the lack of nutritional counselling for the participants attending the clinic was a significant barrier to weight decrease. A similar behavioural weight-loss intervention conducted as an RCT demonstrated significantly reduced weight in overweight and obese adults with serious mental illness (33), and included the addition of a dietietic service. While participants in the mental health exercise physiology clinic in this study had access to a dietitian, it was not compulsory and not all participants took up this option.

The implementation of a mental health exercise physiology clinic in a routine community health centre provides real-world evidence to for the feasibility and acceptability of such clinics in other mental health services. Australia has clear policy directives and guidelines supporting the implementation of physical health programs for people living with mental illness (18, 20, 34), however there has so far not been wide-spread adoption of such programs by community mental health services (35). When people with mental illness engage in physical activity it results in beneficial effects on mental health, physical health, and quality of life (36). Given regular attendance by people living with mental illness, the low-cost nature of implementation, and successful integration into a busy community mental health centre, the positive findings should encourage mental health services to create their own exercise physiology clinics, building on the framework outlined here, whilst considering the challenges encountered and the recommendations that arise from this evaluation.

### Challenges of the Service

Consistent with other reporting in this area, the main challenges associated with establishing the service were centred around financial aspects, staff culture, physical space, and negotiations with management (19). Given that a fully-equipped gym was not possible to implement due to physical limitations of the building and the associated costs, utilising less-frequently used spaces in the community centre was essential. To procure equipment, grant funding and applications for small allocations of funds through the mental health service was necessary. The benefits of accredited exercise physiologists prescribing physical activity and leading interventions for people with mental illness extends beyond clinical relevance, with examples of economic benefits emerging. Deloitte Access Economics reported exercise physiology services within the Australian mental health sector were cost-effective; with each depressive episode prevented through physical activity services delivered by an accredited exercise physiologist resulting in public savings of AUD$10,062 (~USD$7,400) through improvements to productivity and health system expenditures. Emerging analyses show that mental health interventions incorporating physical activity interventions are cost-effective for health services (37–39) and may help to secure funding needed to implement such programs.

### Limitations of the Study

Results should be interpreted considering several methodological limitations. A lack of control group limits the generalisability of the findings. Establishing the clinic under real-world conditions was critical so that as many mental health consumers as possible could participate. The high volume of clients seen in the community mental health centre, lack of physical space and resources, and embedding the clinic within the service over many months, meant that conducting a pragmatic randomised control trial was not possible. Given the need to formally evaluate the success of similar programs in mental health services, future studies should consider more rigorous evaluation methodologies.

Furthermore, the exercise physiology clinic operated under real world conditions, and clinicians needed to ensure that mental health consumers were not unduly inconvenienced, such that outcome measures were not always able to be obtained. This contributed to the numbers of participants that did not have follow-up outcome measures and reduced the data available for analysis. Due to the clinic operating under real-world conditions with consumer-focused clinical services the primary focus, conditions usually implemented within clinical research studies were not strictly adhered to within this evaluation. For example, the mean time between baseline and follow-up measurements were widely variable, and as such additional factors may have influenced changes in outcome measures such as medical illness, medication changes, clinical input from other medical professionals, among others. Such limitations should be considered in future evaluations of real-world clinical services with wide varying follow-up timepoints.

### Recommendations for Future Practise

When implementing physical activity programs in mental health settings best-practise, evidence- based principles should be applied (17, 35). The clinic in this study implemented many evidence-based elements, including individualised programs tailored to participant needs and goals, expert supervision provided by exercise professionals specialising in mental health, routine metabolic monitoring, and regular sessions focused on behaviour change strategies (12, 13). Future studies implementing an exercise physiology clinic for community-based people living with severe mental illness should consider options for improving the rigour of the outcome assessment methodology, collaboration with mental health services and participant retention. Additionally, future services should incorporate well-structured mental health dietetic components alongside exercise physiology services in addition to other interventions such as metformin which have proven successful for weight loss in similar population groups (40, 41). Future studies would also benefit from examining other factors which may be associated with physical activity improvements including psychological well-being, adherence to treatment, reduction in emergency department presentations, and improvements in mental state.

## Conclusion

The implementation of an exercise physiology clinic for consumers within a community-based mental health service was feasible. Such services can improve moderate-to-vigorous physical activity levels and sedentary time for people living with severe mental illness, however further research is needed to determine whether weight reduction and increased fitness levels can also be achieved. When implementing an exercise physiology clinic, programs should be designed in a collaborative approach considering the individual needs of mental health consumers. Given the high level of consumer engagement, potential to improve the physical and mental health of consumers, and cost-effectiveness of physical activity programs, community-based mental health services should consider developing physical activity services lead by exercise professionals as part of routine care.

## Data Availability Statement

The raw data supporting the conclusions of this article will be made available by the authors, without undue reservation.

## Ethics Statement

The studies involving human participants were reviewed and approved by South Eastern Sydney Local Health District (SESLHD) Human Research Ethics Committee. Written informed consent for participation was not required for this study in accordance with the national legislation and the institutional requirements.

## Author Contributions

HF, LE, and OL delivered the intervention. HF, RM, OL, and PW performed data analysis. All authors contributed to the development of the publication.

## Conflict of Interest

The authors declare that the research was conducted in the absence of any commercial or financial relationships that could be construed as a potential conflict of interest.

## Publisher's Note

All claims expressed in this article are solely those of the authors and do not necessarily represent those of their affiliated organizations, or those of the publisher, the editors and the reviewers. Any product that may be evaluated in this article, or claim that may be made by its manufacturer, is not guaranteed or endorsed by the publisher.
